# Identification of QTL for resistance to Mediterranean corn borer in a maize tropical line to improve temperate germplasm

**DOI:** 10.1186/s12870-015-0652-9

**Published:** 2015-11-04

**Authors:** Luis Fernando Samayoa, Rosa Ana Malvar, Michael D. McMullen, Ana Butrón

**Affiliations:** Misión Biológica de Galicia, Spanish National Research Council (CSIC), P.O. Box 28, 36080 Pontevedra, Spain; Plant Sciences Research Unit, USDA-Agricultural Research Service; and Division of Plant Sciences, University of Missouri, Columbia, MO 65211 USA

**Keywords:** Maize, Corn borer, Quantitative trait loci, Insect resistance, Cross validation, *Sesamia nonagrioides*, Marker assisted selection

## Abstract

**Background:**

A QTL mapping study for maize resistance to the Mediterranean corn borer (MCB) was performed with a RIL population derived from the cross B73 × CML103. To develop commercial inbreds of maize resistant to the MCB for use in Europe, it would be useful to transfer resistance from tropical germplasm like the subtropical inbred CML103 to temperate lines. The inbred B73 was chosen as representative of the Stiff Stock heterotic group, a major heterotic group used in hybrid grown in both North American and Europe. The objectives were to study the architecture of genetic factors for resistance to MCB and to check the feasibility of using marker-assisted selection (MAS) for transferring those genetic factors.

**Results:**

Eight quantitative trait loci (QTL) were declared significant for resistance traits and eight QTL were located for agronomic traits. Alleles from CML103 at QTL significant for tunnel length could reduce tunnel length made for MCB in inbred B73 in more than 8 cm; favorable alleles for yield were also found in CML103 and no genetic correlation coefficient between tunnel length and yield was detected.

**Conclusions:**

MAS for transferring resistance genes to corn borer attack from CML103 to B73 could be successful based on cross validation results and a negative effect on yield would not be expected.

**Electronic supplementary material:**

The online version of this article (doi:10.1186/s12870-015-0652-9) contains supplementary material, which is available to authorized users.

## Background

*Sesamia nonagrioides* Lef., commonly called Mediterranean corn borer (MCB), is the most important pest of maize (*Zea mays* L.) in the Mediterranean area [1]. The use of *Bt* hybrids seemed the most efficient method for controlling this pest, but transgenic crops are not authorized in many European countries and are not allowed for organic production [2]. Therefore, breeding for resistance to corn borers based on maize genetic variability for resistance would be valuable to the European and organic seed markets. Also, recent studies have reported a reduction of efficacy as some important pests have evolved resistance to *Bt* [3, 4]. In this context, the stacking of several resistant genes has been proposed as one of the means to delay insect adaptation, and maize natural sources of resistance to stem borers could bring promising genes [5].

In a previous research a collection of 121 inbred lines was evaluated for resistance to MCB in a two-year experiment; the inbred B73 was classified as moderately resistant [6]. B73 is an inbred developed from the Iowa Stiff Stalk Synthetic population with great historic importance to breeders because the hybrid B73 × Mo17 has been widely used and is currently relevant as many commercial inbreds have B73 in their pedigrees [7]. The Iowa Stiff Stalk Synthetic population was constituted by 16 inbred lines resistant to stalk breakage [8] and the borer resistance exhibited by inbreds developed from this population could be consequence of mechanical resistance [9]. However, resistance to MCB attack of the inbred B73 is far from attaining the threshold required by farmers. In a previous study, we looked for quantitative trait loci (QTL) for stem tunneling by MCB in an array of recombinant inbred lines (RIL) developed from the cross B73 × Mo17 [10], B73 and Mo17 are both inbreds with some resistance to MCB attack [6], and limited gains will be obtained by combining resistance factors from both parents. This result is most likely due to common resistance factors among temperate germplasm. In recent studies the subtropical inbred CML103 [11] has shown better performance under MCB attack than B73 (unpublished data). CML103 has also demonstrated high general combining ability; therefore CML103 appears to be a promising candidate to donate novel genes for MCB resistance to temperate germplasm. In this study, QTL analysis for MCB resistance and agronomic traits was performed in a population of RIL derived from the cross B73 × CML103. In addition, the feasibility of using marker-assisted selection (MAS) for transferring those genetic factors was explored by testing the bias of each QTL by cross validation test.

For the first time, QTL for resistance to MCB has been detected in a segregating population derived from a cross between inbreds with high and moderate resistance. Previous studies focused on crosses between moderately resistant and susceptible inbreds (EP39 × EP42), between two inbreds moderately resistant (B73 × Mo17) and between two susceptible inbreds (A637 × EP42) [10, 12, 13]. Results obtained until now have widened our knowledge about the genetic architecture of maize resistance to MCB, but lacked applicability. However, breeders around the world could benefit from the release of a version of B73 with increased resistance to MCB by transferring resistance factors from CML103 because some mechanisms of maize resistance could be common for corn borers [12]. Such resistance mechanisms could include chemical defense systems like benzoxazinoids, mayzin, protease inhibitors, etc. or physical defense traits related with cell wall components like lignin or silica [14].

## Results

Significant difference between B73 and CML103 were found for two resistance traits, tunnel length and stalk damaged (Table 1). Heritabilities for resistance traits ranged from low to moderate while for agronomic traits ranged from moderate to high (Table 1). Moderate genetic correlation between tunnel length and plant height (*r*_*g*_ = 0.63) and high genetic correlation coefficients between tunnel length and stalk damaged (*r*_*g*_ = 0.87) were found (Table 2).

**Table 1:** Means and their standard errors (± SE), and heritabilities (*h* ^2^) of RIL population derived from B73 × CML103 for traits related to resistance to the MCB and agronomic traits evaluated in two years. Mean comparisons of the parental inbreds are also shown

	Resistant traits					Agronomic traits		
	Tunnel length (cm)	Stalk damaged (%)	Kernel resistance (1–9)^a^	Shank resistance (1–9)^a^	Stalk lodging (%)	Yield (Mg ha^−1^)	Silking (days)	Plant height (cm)
RILs								
Mean	39.8	18.5	8.2	7.8	15.4	5.2	89	215
± SE	9.8	4.2	0.4	0.8	13.8	1.8	3	21
*h* ^2^	0.49^b^	0.36^b^	0.35^b^	0.11	0.34^b^	0.69^b^	0.80^b^	0.85^b^
Parents								
B73	53.9	25.3	8.1	6.6	10.1	6.3	85	215
CML103	30.0	13.7	8.4	7.5	35.5	8.7	90	222
LSD (α = 0.05)	12.8	6.6	-	-	-	-	-	-

Heritabilities (*h* ^2^) for each trait were estimated following Holland et al. [15]

^a^Kernel, and shank resistance were scored on a subjective visual scale of 1 to 9 in which 1 indicates completely damaged and 9 indicate no damaged by the larvae

^b^Significantly different from zero at 0.05 probability level

**Table 2:** Genotypic and phenotypic correlation coefficients and their standard errors between tunnel length by the MCB and other traits recorded in a collection of RILs derived from B73 × CML103 evaluated in a two-year experiment under artificial infestation

	Phenotypic correlation	Genotypic correlation
Stalk lodging	−0.11 ± 0.04	−0.11 ± 0.22
Days to silking	0.04 ± 0.04	0.03 ± 0.13
Plant height	0.21 ± 0.04	0.63 ± 0.12
Yield	−0.05 ± 0.04	0.03 ± 0.14
Stalk damaged	0.94 ± 0.01	0.87 ± 0.04
Kernel resistance	−0.21 ± 0.04	0.07 ± 0.22

The genetic map covered a length of 1388.5 cM. The average interval between markers was of 10.1 cM. No segregation distortion from the expected ratio was observed in the analyses for any marker.

**Fig. 1:**
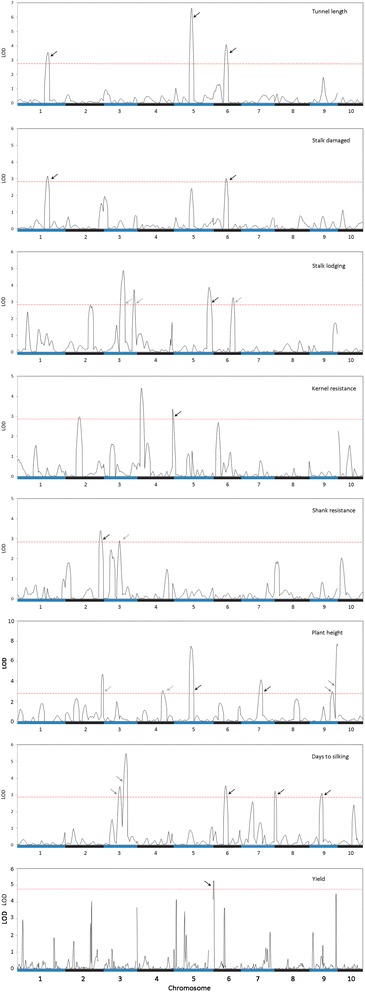
Whole-genome scans to detect QTL for resistance and agronomic traits. Solid black line represents the LOD curve obtained with QTL scan using a marker interval of approximately 10 cM and the red dashed line indicates the LOD threshold chosen by permutation test to declare the presence of a significant. Gray arrows indicate the presence of putative QTL which were detected in the preliminary fit but not in the final fit of the model selection. Black arrows indicated the QTL which were included in final fit of the QTL analysis

In a preliminary fit of the model selection, putative QTL for stalk lodging, kernel resistance, shank resistance, plant height, and days to silking were identified and mapped to different genetic positions (indicated by a gray arrow as suggestive QTL in Fig. 1). The LOD peaks that exceeded the LOD threshold chosen by permutation test (for each trait) indicated the presence of putative QTL (Fig. 1) but several of them were excluded after a final fit of the model controlled by the Bayesian information criterion (BIC) was performed. For tunnel length, days to silking, and yield under infestation with MCB, all the LOD peaks that exceed the previously fixed LOD threshold were selected as real QTL in the final fit.

**Fig. 2:**
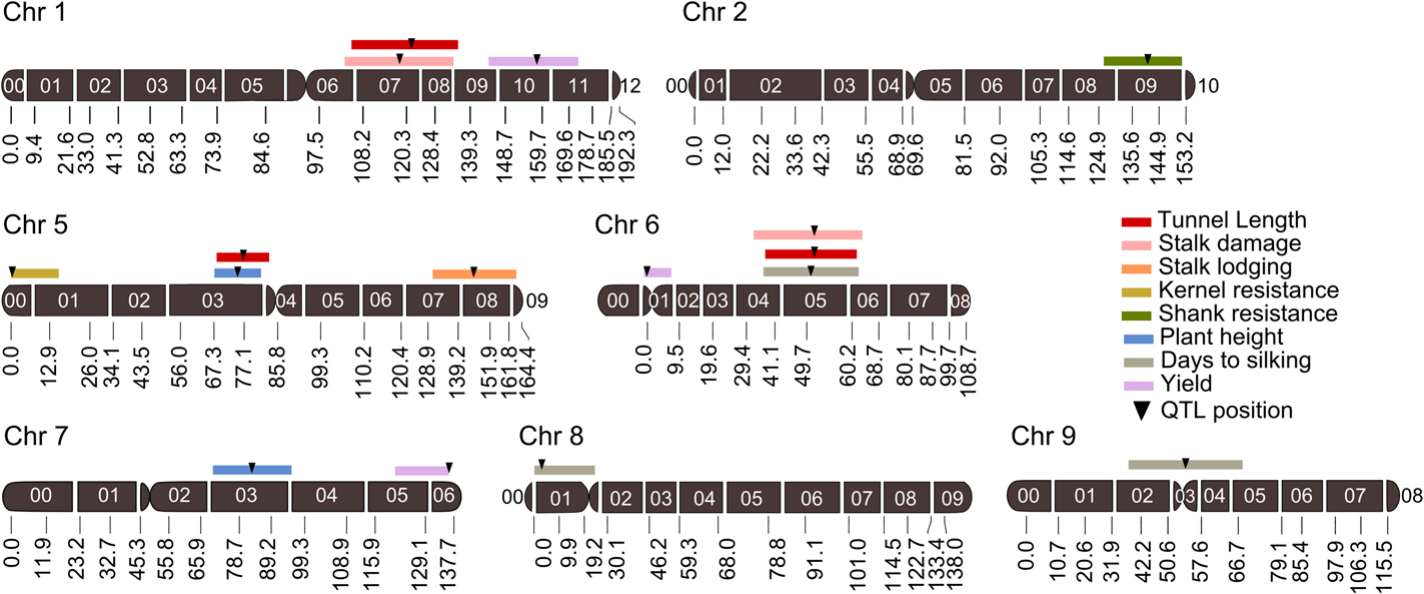
Molecular linked map of maize based on 147 SNP markers and QTL positions. The genetic map was constructed on 178 RILs derived from the B73 × CML103. Only those chromosomes where QTL were located are shown. The black number aligned below each chromosome indicated the position in cM of each SNP marker and white numbers aligned on each chromosome indicate the bin number. 95 % confidence intervals are indicated by the length of QTL bar

Eight QTL for resistance traits were identified in this RIL population (Table 3, Fig. 2). Three QTL for tunnel length were located on chromosome 1, 5 and 6 and accounted for more than 50 and 25 % of the total genetic and phenotypic variance, respectively, with a percentage of estimation bias between 22 and 67 %. The additive effects ranged from 3 to 4 cm for each QTL with a bias estimation between 6 and 45 %. For stalk damaged two QTL were located on chromosome 1 and 6, each explained more than 30 and 10 % of the genetic and phenotypic variance, respectively, but the estimation biases for those parameters were too high (>95 %). The additive effect estimated in the test set (${\overline{\alpha}}_{TS}$, more detailed explanation is in methods section) for both QTL for stalk damage were less than 1 %. A QTL for stalk lodging was located on chromosome 5 which accounted for 17 and 5.8 % of the genetic and phenotypic variance, respectively, with an estimation bias higher than 90 %. The absolute value of the additive effect estimated in the test set (TS) for this QTL was 2.2 %. Another QTL on chromosome 5 was located for kernel resistance which accounted for 10 and 3.5 % of the genetic and phenotypic variance, respectively. However the estimation of these parameters was completely biased as suggested by the results of the cross validation (CV) test (Table 3). In addition, the additive effect estimated in the TS was very small (0.02 point in the subjective scale from 1to 9). For shank resistance, one QTL of small additive effect (${\overline{\alpha}}_{TS}$ = 0.19) was located on chromosome 2. This QTL explained 66 and 7 % of the genetic and phenotypic variance, respectively, with an estimation bias of 58 %. Eight QTL were identified for agronomic traits (Table 3, Fig. 2). Two QTL for plant height were located on chromosome 5 and 7. These QTL accounted for 23 % and 19 % of the total genetic and phenotypic variance, respectively, and the bias estimation of these parameters was of 38 and 82 % for the QTL on chromosomes 5 and 7, respectively. The absolute value of additive effect estimated in the TS was 8.31 cm for the QTL on chromosome 5 and 5.7 cm for the QTL on chromosome 7. The detection frequency of the QTL on chromosome 5 was higher (0.90) than that observed in the QTL of chromosome 7 (0.38).

**Table 3:** Summary of QTL mapped in the RIL population derived from B73 × CML103 under MCB infestation using a genetic map with an average interval between markers of 10 cM

QTL position					Genetic variability explained (%)				Additive mean effect^e^					
bin^a^	cM	95 % CI^b^ (cM)	LOD^c^	Flanking markers’ positions (bp)	DS^d^	ES^d^	TS^d^	Bias^d^	DS	ES	TS	Bias	Freq^f^	$\left({\widehat{R}}^2\right)$ ^g^
Tunnel length (cm)														
1.07-1.08	123	107-139	3.53	218577918-235278541	13.22	17.39	5.66	0.67	−3.00	−3.46	−1.91	0.45	0.49	6.5
5.03	77	68-86	6.63	30460922-73132746	24.14	21.57	16.77	0.22	−4.06	−3.88	−3.66	0.06	1.00	11.8
6.05-6.06	52	38-66	4.08	147913712- 156011668	14.83	18.11	9.82	0.46	−3.12	−3.60	−2.64	0.27	0.69	7.3
Stalk damaged (%)														
1.07-1.08	121	103-139	3.16	218577918-235278541	18.09	25.64	1.01	0.96	−1.15	−1.47	−0.63	0.57	0.26	6.5
6.05-6.06	52	34-70	3.01	147913712-156011668	14.62	21.84	0.42	0.98	−1.06	−1.34	−0.51	0.62	0.24	5.3
Stalk lodging (%)														
5.07-5.08	149	135-163	3.88	211274389-213564164	16.97	20.16	1.89	0.91	−4.08	−4.47	−2.2	0.51	0.41	5.8
Kernel resistance (1-9)^h^														
5.00-5.01	0	0-16	3.37	417591-3366862	10.1	15.09	−2.97	1.20	0.10	0.13	0.02	0.85	0.20	3.5
Shank resistance (1-9)^h^														
2.09	142	126-153	3.41	230206347-233622738	66.17	70.9	29.48	0.58	0.25	0.26	0.19	0.27	0.73	7.3
Plant height (cm)														
5.03	75	67-83	7.51	30460922-73132746	15.64	15.29	9.48	0.38	−8.91	−8.79	−8.31	0.05	0.90	13.3
7.03	83	70-96	4.15	135637466-153719657	7.09	8.59	1.52	0.82	−5.70	−6.29	−2.51	0.60	0.38	6.0
Days to silking														
6.05-6.06	51	35-67	3.55	147913712-156011668	5.34	8.42	1.35	0.84	0.62	0.83	0.39	0.53	0.29	4.3
8.00-8.01	3	0-20	3.22	578045-4383913	7.32	9.1	0.53	0.94	−0.78	−0.87	−0.30	0.66	0.28	5.9
9.02-9.04	53	35-71	3.09	20223300-110404915	11.59	12.67	3.95	0.69	0.99	1.04	0.74	0.29	0.55	9.3
Yield (Mg ha^−1^)														
1.10	159	144-174	3.69	274576136-281980858	8.43	10.43	2.23	0.79	0.43	0.48	0.21	0.56	0.34	5.9
6.01-6.02	1	0-9	6.74	9498146-88522572	16.76	17.11	12.01	0.30	−0.64	−0.60	−0.58	0.03	0.95	11.7
7.05-7.06	137	119-137	3.03	172883402-176785230	7.4	10.25	0.4	0.96	−0.40	−0.47	−0.06	0.87	0.17	5.2

^a^Bin locations are designed by an X.Y code, where X is the linkage group containing the Bin and Y is the location of the Bin within the linkage group [16] . Bins were based on the physical position of flanking markers

^b^95 % confidence interval as explained in Utz (17)

^c^LOD score in the LOD-profile used in scanning for QTL

^d^DS, estimation in the complete data set; ES, average values of the 1000 estimation sets (80 % of the genotypes of DS) in cross-validation; TS, average values of the 1000 validation sets (20 % of the genotypes of DS) in cross validation; Bias, estimation bias calculated as the difference between ES and TS estimations divided by ES estimation

^e^Additive effect of the QTL estimated as half the difference between the genotypic values f the two homozygotes. A positive estimation means that CML103 carries the allele with higher value

^f^Frequency in cross-validation of QTL found within the 1-LOD support interval

^g^ Proportion of phenotypic variance which is explained by the QTL

^h^subjective visual resistance scale of 1 to 9 in which 1 indicates completely damaged and 9 indicate no damaged by the larvae

Three QTL were detected for days to silking on chromosome 6, 8, and 9. The proportion of the genetic and phenotypic variance explained by the three QTL was 24.2 and 19.5 %, respectively, with an overestimation from 69 to 94 %. The additive effect estimated in the TS for each QTL was less than 1 day. For yield, three QTL were located on chromosomes 1, 6, and 7. The proportion of genetic and phenotypic variance explained by the three QTL was 33 and 23 % and the estimation bias of the genetic variance explained by each QTL ranged from 30 to 96 %. The additive effect estimated in TS ranged from 0.1 to 0.6 Mg ha^−1^. The detection frequency through the CV runs was of 34, 95, and 17 % for the QTL in chromosomes 1, 6, and 7, respectively.

## Discussion

The heritabilities observed for tunnel length by MCB was intermediate and compare favorably to those obtained with other RIL populations under similar conditions of infestation with MCB [10, 12]. Heritability for kernel resistance observed herein (*h*^2^ = 0.35) is inferior to that obtained (*h*^2^ = 0.5) by Ordás et al. [10]. In addition, the heritability for shank resistance was not different from zero which is in agreement with Samayoa et al. [12]. The heritabilities for agronomic traits were similar to those obtained by other authors in numerous, diverse RIL populations [18–20].

As the goal is to detect reliable QTL, most discussion will be focused on results from the final fit of the model selection that is conditioned by the Bayesian information criterion (BIC) [17]. Although this model selection criteria tend to find slightly fewer QTL compared with other criteria it minimizes the risk of selecting spurious QTL [21].

No QTL for stalk tunneling by MCB were previously reported in bins 1.07-1.08, 5.03, and 6.05-6.06 [10, 12, 13, 22]. However, QTL for tunnel length by the European corn borer (ECB, *Ostrinia nubilalis*) have been previously mapped to chromosomes 1 and 5 [18, 23–25]. Krakowsky et al. [26] and Orsini et al. [27] also localized QTL for tunnel length and stalk breakage by ECB in the bin 6.05. As all favorable alleles for tunnel length in this study came from the subtropical line CML103, this inbred could clearly enhance the resistance of the line B73 by providing new alleles of resistance in chromosomes 1 and 5 and, even, in the chromosome 6 where it is known that the B73 line carries alleles associated with resistance to tunnel length by ECB [26]. In addition, the additive effects for the three QTL detected for tunnel length were, in general, higher ($\widehat{\alpha}$ = 3–4.1 cm) than those reported in the studies mentioned above ($\widehat{\alpha}$ = 0.5-1.2 cm) and, most importantly, the CV analysis revealed that the reliability of QTL for tunnel length was moderate to high.

QTL for tunnel length and plant height were co-localized in the same region of the chromosomes 5. In addition, a significant and moderate genetic correlation between tunnel length and plant height was found agreeing with results of previous QTL studies with artificial infestation both with MCB [12, 13] as with ECB [24, 28, 29]. In addition, in a recent association mapping for resistance to MCB attack it was observed an intermediate and positive genetic correlation between tunnel length and plant height but no significantly associated SNP was co-localized for both traits [22], therefore it remains necessary to carry out deepest studies to elucidate if these findings are due to linkage or pleiotropy. QTL for tunnel length and days to silking were also co-localized on chromosome 6. Opposite signs of the additive QTL effects for these traits indicate that flowering time could be slightly delayed when transferring resistance alleles from CML103 to B73 but it was not supported by the genetic correlation between these traits. Yield would not be significantly modified because no genetic correlation was found between tunnel length and yield under infestation unlike to other studies in which selection to reduce tunnel length made by corn borers has resulting in an important reduction of yield probably due to linkage between certain alleles for resistance and some alleles affecting maize yield. [30–32].

The final fit for days to silking revealed the presence of three QTL in chromosomes 6, 8 and 9; one of them was also detected by Buckler et al. [33] in the same RIL population and genotyping data but different data analysis method. The QTL for days to silking on chromosomes 8 and 9 detected herein were not detected by those authors; while a QTL in chromosome 3 detected by Buckler et al. [33] was found in the preliminary fit but it was not retained in the final fit. These discrepancies between our results and those provided previously by Buckler et al. were probably due to QTL × environment interaction effects and stressed the importance of making phenotypic evaluations in environments similar to those for which breeding materials are intended.

We identified three novels QTL for grain yield under infestation with *S. nonagrioides* in chromosomes 1, 6, and 7. In previous studies, QTL for yield under infestation with MCB were located on chromosomes 4, 5, and 8 [12, 13], and QTL for grain yield under infestation with ECB have been reported on chromosomes 2, 4, 6, 8, 9, and 10 [18–20]. Alleles from the line B73 at QTL in chromosomes 6 and 7 increased grain yield, but the allele from CML103 for the QTL in chromosome 1 could be used to improve yield of the inbred B73. Although this QTL would need to be tested in hybrid for efficacy since yield QTL in inbreds versus hybrids are poorly correlated. In general, the additive effects estimated in TS of each of the three QTL (0.1 – 0.6 Mg ha^−1^) were higher than those obtained by authors mentioned above (0.2 – 0.3 Mg ha^−1^). The QTL located in chromosome 6 is especially interesting because it explained a high proportion of the genetic variance (17 %) with the lowest estimation bias for this parameter and with a high detection frequency (95 %) through CV runs. Although no QTL for yield under infestation with corn borers have been previously found in this region (bin 6.01- 6.02) several studies have reported important QTL for grain yield and its components in physiological conditions in the same region in other mapping populations [34–38]. Even the results of a fine mapping study suggest that a pleiotropic locus could be affecting grain yield and related traits in this region of chromosome 6 [39].

## Conclusion

The inbred CML103 could enhance the resistance of the inbred B73 without reducing its yield under infestation by providing new alleles of resistance in chromosomes 1, 5 and 6 where it is known that the inbred B73 carries alleles associated with resistance to tunnel length by ECB. The inbred CML103 could also potentially provide favorable alleles for yield under infestation with MCB on chromosome 1.

Three novels QTL for yield under infestation with MCB were found in this RIL population, the highly reliable QTL of chromosome 6 with an additive effect of 0.6 Mg ha^−1^ being particularly important.

Cross validation analyses confirmed the moderate to high reliability of QTL detected for tunnel length and supported the use of markers associated to these QTL for performing marker-assisted selection in order to transfer resistance alleles from CML103 to B73.

## Methods

The 178 RILs obtained from the cross B73 × CML103 are part of the nesting association mapping population with genotype at 1478 SNPs provided [40, 41]. Based on preliminary analysis with all the markers and the conclusion of previous research comparing the use of high vs. low density marker map [42, 43] we constructed a genetic map using MAPMAKER software with a subset of 147 markers (see Availability of supporting data section) to obtain an average marker interval of 10 cM. One hundred percent of the genome was within 20 cM of the nearest marker in the genetic map.

The 178 RILs were evaluated in 2011 and 2012 along with the parental inbreds B73 and CML103 using a 14 × 14 single lattice design with two replication per year. The trials were hand planted and each experimental plot consisted of one row spaced 0.8 m apart with 13 two-kernel hills spaced 0.18 m apart. Plots were overplanted and thinned, obtaining a final density of ~70,000 plant ha^−1^. The evaluations were performed under artificial infestation with MCB eggs obtained at the Misión Biologica de Galicia by rearing the insect as described by Eizaguirre and Albajes [44] and Khan and Saxena [45]. Before flowering, five plants of each plot were infested with ~40 MCB eggs placed between the stem and the sheath of a basal leaf. Data collected were (Additional file 1): days to silking as the days from planting to the date 50 % of plants showing silks; plant height as the average length (in cm) from the ground to the top of five representative plants; stem lodging defined as the percentage of plants in the plot with the stem broken below the main ear; kernel resistance and shank resistance to MCB larvae measured on the ears of the five infested plants according to a subjective visual resistance scale of 1 to 9 in which 1 indicates completely damaged and 9 indicates no damage; tunnel length as the average length in cm of stem tunnels made by borers on the five infested plants; the percentage of stem damaged by the larvae calculated as tunnel length divided by plant height and multiplied by 100; and kernel yield estimated on a plot basis as Mg ha^−1^ at 140 g H_2_O kg^−1^.

Individual phenotypic data (per year) was analyzed in SAS software using the mixed model procedure (PROC MIXED) [46] considering replications and blocks within replications as random effects and RILs as fixed effects. Then, combined analysis across year was conducted considering RILs as the only fixed effects. A best linear unbiased estimator (BLUE) was obtained to estimate each line mean phenotypic value both for individual as for combined data. Heritabilities (*ĥ*^2^) across environments were estimated for each trait on a family-mean basis as described by Holland et al. [15]. The genetic (*r*_*g*_) and phenotypic (*r*_*p*_) correlations between traits were computed following Holland [47]. All previous analyses were made in SAS software version 9.4 (see Additional file 2 for more details of code).

QTL analysis was performed using the software package PlabMQTL [17]. Composite interval mapping approach was conducted for QTL detection and to estimate QTL effects using the command cov SEL (Additional file 2). According to a previously executed permutation test with 1000 random reshuffles [48], LOD thresholds of 2.9 (with and empirical critical value of 25 %) were chosen to declare significant the presence of a putative QTL. The QTL mapping was conducted by a two-step procedure: in a first step an entire genome scanning is performed to draw the LOD curves and identify the peaks where the putative QTL are located, in this preliminary fit are estimated the additive effects of all preselected cofactors. In the second step the more important genetic effects of QTL of previous step are screened using the BIC as criteria of selection during the stepwise regression procedure [17]. Following Utz et al. [49], a five-fold cross validation (CV) approach was employed for obtaining unbiased estimation of the QTL parameters such as genetic ($\widehat{p}$) and phenotypic (${\widehat{R}}^2$) variance explained by each putative QTL and its respective additive effect ($\widehat{\alpha}$). For each trait, CV was performed for the whole data set (DS) of entry BLUEs across environments. A total of 142 entries were used as estimation set (ES) for calibration and 36 entries were used as the test set (TS) for validation. One thousand CV runs were performed in order to determine the QTL frequency and shrinkage of estimations for QTL effects and proportion of the genetic and phenotypic variance explained by the QTL detected in the original data set [50]. The magnitude of the bias of the estimation of ${\widehat{p}}_i$ explained by each individual QTL *i* was calculated as the difference between the average estimate of $\overline{p}$ obtained in ES and the corresponding estimate in TS (${\overline{p}}_{i\ ES} - {\overline{p}}_{i\ TS.ES}$) divided by ${\overline{p}}_{i\ ES}$. In the same way the biases for the estimates of additive effects ${\widehat{\alpha}}_i$ were obtained. A bias of 50 % was established as cutoff to consider low or high the estimation bias of each parameter. The Grep utility [51] was employed to extract, in each CV run, the proportion of genotypic (${\widehat{p}}_{i\ ES}$ and ${\widehat{p}}_{i\ TS}$) and phenotypic (${\widehat{R}}_{i\ ES}^2$ and ${\widehat{R}}_{i\ TS}^2$) variances of the ES and TS explained by each individual QTL *i* detected and also the additive effects (${\widehat{\alpha}}_{i\ ES}$ and ${\widehat{\alpha}}_{i\ TS}$).

### Availability of supporting data

Genotypic data of a RIL population derived from B73 × CML103 used in QTL analysis in this research are available in the Digital CSIC repository in http://hdl.handle.net/10261/123685. This array was taken from the NAM population [40, 41] genotyping data set (phased and fully imputed genotypes at 1 cM resolution) available in http://mirrors.iplantcollaborative.org/browse/iplant/home/shared/panzea/genotypes/GBS/v23/NAM_phasedImputed_1cM_AllZeaGBSv2.3_allChrs.zip.

## Additional files

Additional file 1:
**Phenotypic data of a RIL population derived from B73 × CML103.** Data set containing the result of the evaluation of resistance and agronomic traits in two year with two replications per year at Pontevedra, Spain. (XLSX 73 kb) Additional file 2:
**Code used in phenotypic statistical analysis and QTL analysis.** SAS code used to estimate BLUE, heritabilities and genotypic and phenotypic correlation. PlabMQTL code used in QTL analysis. (TXT 13 kb)

## Abbreviations

BIC: Bayesian information criterion
BLUE: best linear unbiased estimator
CV: cross validation
DS: data set
ECB: European corn borer
ES: estimation set
MAS: marker assisted selection
MCB: Mediterranean corn borer
QTL: quantitative trait locus/loci
RIL: recombinant inbred lines
SNP: single nucleotide polymorphism
TS: test set
